# *In silico* error correction improves cfDNA mutation calling

**DOI:** 10.1093/bioinformatics/bty1004

**Published:** 2018-12-06

**Authors:** Chang Sik Kim, Sumitra Mohan, Mahmood Ayub, Dominic G Rothwell, Caroline Dive, Ged Brady, Crispin Miller

**Affiliations:** bty1004-aff1 Clinical and Experimental Pharmacology Group, Cancer Research UK Manchester Institute; bty1004-aff2 Medical Research Council Manchester Single Cell Research Centre; bty1004-aff3 RNA Biology Group, Cancer Research UK Manchester Institute, University of Manchester, Alderley Park, Manchester, UK

## Abstract

**Motivation:**

Circulating-free DNA (cfDNA) profiling by sequencing is an important minimally invasive protocol for monitoring the mutation profile of solid tumours in cancer patients. Since the concentration of available cfDNA is limited, sample library generation relies on multiple rounds of PCR amplification, during which the accumulation of errors results in reduced sensitivity and lower accuracy.

**Results:**

We present PCR Error Correction (*PEC*), an algorithm to identify and correct errors in short read sequencing data. It exploits the redundancy that arises from multiple rounds of PCR amplification. PEC is particularly well suited to applications such as single-cell sequencing and circulating tumour DNA (ctDNA) analysis, in which many cycles of PCR are used to generate sufficient DNA for sequencing from small amounts of starting material. When applied to ctDNA analysis, *PEC* significantly improves mutation calling accuracy, achieving similar levels of performance to more complex strategies that require additional protocol steps and access to calibration DNA datasets.

**Availability and implementation:**

*PEC* is available under the GPL-v3 Open Source licence, and is freely available from: https://github.com/CRUKMI-ComputationalBiology/PCR_Error_Correction.git.

**Supplementary information:**

Supplementary data are available at *Bioinformatics* online.

## 1 Introduction

Next Generation Sequencing (NGS) of circulating tumour DNA (ctDNA) from patient blood has the potential to revolutionize cancer genomics by supporting non-invasive tumour genotyping using blood-based ‘liquid’ biopsies (Bettegowda *et al.*, 2014; Bratman *et al.*, 2015; Butler *et al.*, 2015; Diaz and Bardelli, 2014; Diehl *et al.*, 2008; Heitzer *et al.*, 2015; Kurtz *et al.*, 2015). It is challenging because ctDNA accounts for as little as 0.01% of the total circulating free DNA (cfDNA) population (Bettegowda *et al.*, 2014; Bratman *et al.*, 2015; Newman *et al.*, 2014; Schmitt *et al.*, 2012). Reliable variant detection therefore requires high sequencing depth and many rounds of amplification. This results in elevated PCR error rates and reduced mutation calling accuracy. PCR errors arise from a variety of sources including DNA damage, structure induced template-switching, PCR-mediated recombination and polymerase misincorporation (Potapov and Ong, 2017). Together, these lead to a mixture of systematic and random changes to the amplified molecular sequence.

A number of methodologies have been developed to correct PCR errors (Kennedy *et al.*, 2014; Newman *et al.*, 2014, 2016; Schmitt *et al.*, 2012), including Duplex Sequencing (DS) (Kennedy *et al.*, 2014; Newman *et al.*, 2014; Schmitt *et al.*, 2012), which relies on barcodes to track the PCR products arising from each DNA fragment. This allows them to be grouped together, and the consensus between their sequences used to identify and correct errors.

A recently developed method, *iDES* (Newman *et al.*, 2016), uses DS in combination with a second ‘*background-polishing*’ step that corrects systematic errors found preferentially within certain sequences. These have the potential to act as a major confounding factor, since they can accumulate across specific loci, mimicking a *bona fide* variant allele. To do this, *iDES* uses a background model built from healthy normal volunteer (HNV) blood samples to identify ‘hotspot’ regions with disproportionately high error rates. Variants at these loci are then filtered from the results. While a significant advance, *iDES* requires high sequencing depths, access to HNV datasets, and an additional barcoding step. These add cost and complexity to the protocol, and may to reduce the efficiency of library preparation—particularly undesirable when dealing with limited amounts of ctDNA material. A similarly sensitive and barcode-free approach that does not require extremely high-depth sequencing and external HNV calibration data would therefore further advance the field.

Here, we describe PCR Error Correction (*PEC*), a new algorithm for short-read sequencing data. *PEC* uses an *in silico*, barcode-free, strategy to exploit the redundancy that arises when multiple PCR amplicons are derived from the same initial DNA fragment. Since these duplicate reads can confound statistical analyses, they are typically discarded following alignment, leaving only a single read, presumed to represent the original cfDNA molecule. Typically, the terms ‘Sequencing Depth’ and ‘Read Depth’ are used to refer to the number of overlapping reads before and after de-duplication. For the purposes of this manuscript, it is useful to define a third term ‘Duplicate Depth’ to refer to the number of PCR duplicates in an amplicon cluster.

De-duplication is commonly done using the *MarkDuplicates* algorithm from Picard (DePristo *et al.*, 2011). *MarkDuplicates* relies on the fact that at typical sequencing depths, the likelihood of two read fragments originating from exactly the same genomic region is negligible (Methods). It therefore identifies all reads with the same 5' end (taking into account strandedness) and retains the read with the highest overall base quality score. While this deals well with errors resulting from low-quality sequencing, it makes no attempt to identify high quality reads that nevertheless incorporate PCR errors. These are then able to propagate to subsequent stages of the analysis pipeline.

**Fig. 1.**
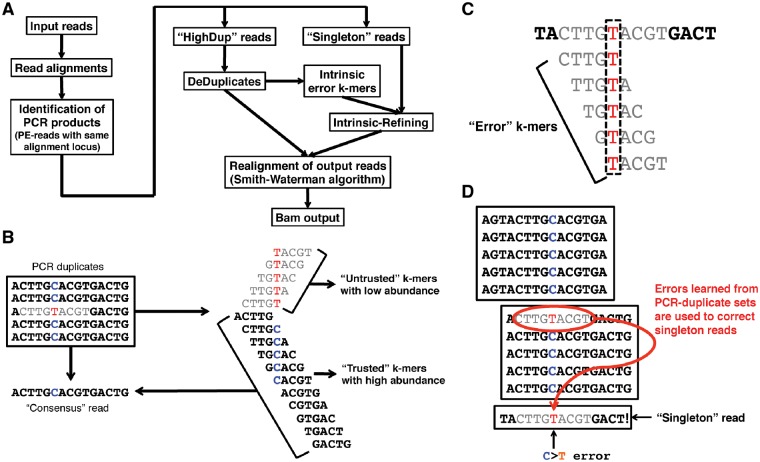
PCR Error Correction (*PEC*) algorithm. (**A**) Overview of PEC algorithm pipeline design. *DeDuplicates*: de-duplication using *de novo* assembly, in which consensus reads and error k-mer lists are identified. (**B**) Each PCR duplicate set is independently assembled using a k-mer assembler (based on a *de Brujin* graph) to produce a consensus sequence. K-mers that do not match this consensus are flagged as ‘untrusted’, while those that match the consensus are flagged as ‘trusted’. (**C**) Untrusted k-mers that have never been flagged ‘trusted’ are considered to be error k-mers. These are used to identify errors in reads that are in low coverage PCR amplicon sets with insufficient duplicate depth to allow a reliable consensus sequence to be generated. (**D**) In this way, error patterns learnt from correcting errors in high-coverage PCR amplicon sets are used to correct errors in low-coverage and singleton reads

*PEC* first identifies reads expected to arise from the same initial starting molecule, using a similar strategy to *MarkDuplicates.* However, rather than retaining the read with highest quality score, it instead uses a local sequence assembly to generate the consensus sequence for each PCR amplicon set (Fig. 1A). While doing this, it learns the ‘intrinsic error pattern’ within the data, comprising the most frequently corrected sequence patterns. This is then used to identify and correct systematic errors in singleton reads. Here, we show that this results in equivalent performance to *iDES*, but without the need for a reference HNV control dataset or an additional *in vitro* barcoding step.

## 2 Materials and methods

### 2.1 The *Pec* algorithm

*PEC* is implemented using the MapReduce-MPI C++ library (Plimpton and Devine, 2011), an open-source implementation of the MapReduce framework written for distributed memory parallel machines on top of the open Message Passing Interface (MPI) library. The overall pipeline can be parallelized across multiple processors. It proceeds through several steps (Fig. 1A). The first identifies and groups input reads that are likely to have been amplified from the same cfDNA molecules. *PEC* assumes that paired-end reads aligning to the same genomic location (by 5' end) are PCR products derived from the same initial DNA fragment. This is similar to the approach taken by *MarkDuplicates* (DePristo *et al.*, 2011). Locations are identified by aligning reads to the reference genome using *BWA* (Li and Durbin, 2009). Each read is represented by a key-value pair in a MapReduce implementation, with the genomic locations providing the keys. Paired-end reads can then be clustered by a single MapReduce step to produce sets of PCR duplicates, each with the same genomic locations. It then performs two error suppression steps. The first, *DeDuplicates*, applies only to sets with a relatively high number of PCR duplicates (duplicate depth ≥ 5 by default; note that this refers to the number of read-pairs in the PCR duplicate set, not the read depth following de-duplication).

A linear sequence assembly algorithm (Grabherr *et al.*, 2011) is applied to each set to generate its consensus sequence. At each nucleotide in the sequence, the most common allele is selected at the representative nucleotide. Since the assumption is that all read-pairs mapping to the same 5' coordinates are likely to have originated from the same DNA fragment, this serves to identify and correct PCR errors (Fig. 1B).

In parallel*, PEC* generates, for each set, the list of k-mers used in the alignment. These are marked as ‘trusted’ or ‘untrusted’, such that ‘trusted’ k-mers match the consensus sequences, while ‘untrusted’ k-mers include a potential error residue. Note that error bases with a low base calling quality score are not considered to be PCR errors (Phred quality score ≥20 by default). Since these have already been identified as potential errors by the scanner software, PEC leaves them unchanged. Downstream analysis software is then able to deal with them in the usual way.

The k-mer lists from each PCR-amplicon set are then merged in order to generate a list of ‘error’ k-mers, comprising all ‘untrusted’ k-mers that have never been flagged as ‘trusted’. This final list is then applied only to singleton reads and PCR-amplicon sets with few PCR duplicates (duplicate depth < 5 by default). For these, the paired-end read with the highest overall base quality scores is selected as the representative cfDNA molecule for each amplicon set. Candidate error bases are then localized by the intersection of error k-mers (Kelley *et al.*, 2010) (Fig. 1C) and flagged within the representative reads by lowering the base quality score for that nucleotide by setting the Phred score to 5 for that base, allowing them to be ignored in subsequent downstream analyses (Fig. 1D). Since this second step learns error patterns from the dataset itself, rather than an external reference set, we refer to it as *Intrinsic-Polishing*. NGS aligners, such as BWA employ some degree of compromise between speed and accuracy. The performance of BWA also begins to degrade for longer reads and/or those with high error rates (Li and Durbin, 2009). Since the de-duplicated dataset produced by PEC is significantly smaller, and thus more computationally tractable, PEC employs a final step in which the de-duplicated reads are realigned to the reference genome using the Smith-Waterman C/C++ library (Zhao *et al.*, 2013). This then generates the optimal alignment for each read. The output of this software is an error-corrected set of cfDNA reads in bam file format, which can be easily incorporated into existing NGS data analysis pipelines.

### 2.2 Likelihood of cfDNA collisions

The strategy employed by PEC is dependent on individual ctDNA molecules originating from distinct position on the genome. Here we justify this assumption, using a derivation based on the Birthday Paradox.

The probability *C_L_* of having at least one cfDNA collision at the given locus can be defined as follows:

(1) $$C_{L}=1-\frac{M_{L}!\left(\begin{array}{c} L\\ M_{L} \end{array}\right)}{L^{M_{L}}}$$

where M_L_ represents the expected number of cfDNAs of fixed length L at a given locus.

In a real experiment, cfDNA fragments are of different lengths. Since a collision occurs when two cfDNA fragments have identical start and end points, cfDNA fragments with different lengths cannot collide. We therefore model the fragment length distribution using a normal distribution with mean μ, σ, to produce a distribution for M_L_, given the cfDNA depth, D:

(2) $${\hat{M}}_{L}=D\times N(L|\mu ,\sigma )$$

Third, the probability p of having any cfDNA collisions at each locus with varying length L can be defined as,

(3) $$p=\int C_{L}N\left(L|\mu ,\sigma \right)dL=\sum_{L}C_{L}N\left(L|\mu ,\sigma \right)$$

With cfDNA prepared using the protocol described in Section 2.3.4, below, typical values of μ=177,σ=20 were determined using an Agilent Bioanalyzer (data not shown). Using Equation (3), and these values, p=0.047 with D = 300*X*, i.e. ∼4.7% of the target region is expected to have cfDNA collisions (Supplementary Fig. S1).

While these are typical values for ctDNA library preparation, much greater sequencing depths, longer fragments, or less variable fragment sizes may increase the likelihood of collisions. We therefore recommend computing *p* to confirm that this collision assumption holds, if the library preparation step is expected to generate a dramatically different distribution. For example, once the cfDNA depth (as distinct from the sequencing depth) exceeds 500× then the likelihood of collision becomes prohibitive.

### 2.3 Evaluation datasets

#### 2.3.1 Dataset 1

We used a previously published cfDNA-seq dataset (Newman *et al.*, 2016), comprising 4 samples of cfDNAs with simulated ctDNAs (HD500; Horizon Diagnostics 500), a DNA diagnostic reference standard consisting of multiple clinically relevant variants with a range of known allele fractions between 0.94 and 32.5%. The 4 sample replicates were created with 5.0% dilution of acoustically shorn HD500 genomic DNA fragments added to cfDNAs from a healthy donor.

#### 2.3.2 Dataset 2

We simulated the typical cfDNA nucleosomal fragmentation pattern by applying a double-strand DNA specific endonuclease (EZ Nucleosomal DNA Prep Kit, Zymo Research, Orange, CA) as per the manufacturer’s instructions to generate nucleosomal DNA fragments from both a well-characterized SCLC cell line H446 and non-cancer control peripheral blood mononuclear cells (PBMCs). Subsequently we prepared serial dilutions of the nucleosome fragmented DNA to generate PBMC/H446 mixtures where the H446 DNA was present at 100, 20, 10, 5 and 0%. These dilution samples were further NGS whole genome library prepped at final concentrations of 20 and 5 ng to mirror the input cfDNA concentrations from patient samples for NGS whole genome library preparation. The whole genome libraries were enriched for 15 genes using a SureSelectXT Custom DNA Kit (Agilent, Santa Clara, CA, USA) of 15 genes. The enriched DNA libraries were re-amplified using the KAPA HiFi PCR Kits and Illumina sequencing primers for 13 cycles. Paired end sequencing was performed for these enriched libraries on the Illumina^®^ NextSeq 500 (Illumina, San Diego, CA, USA) benchtop sequencer with the NextSeq 500/550 Mid Output v2 kit (300 cycles).

#### 2.3.3 Non-Cancer volunteer sample collection

Blood samples for cfDNA analysis were collected in a Cell-Free™ DNA BCT tubes (Streck, Omaha, NE). The blood samples were transferred to the Clinical and Experimental laboratory for processing. Samples were collected from a non-cancer control volunteer (referred to as HNV or healthy normal volunteer and these were persons recruited from within the CR-UK Manchester Institute that was not currently suffering or being treated for cancer).

#### 2.3.4 Circulating cell free DNA preparation and quantification

Blood samples collected using Cell-Free™ DNA BCT tubes (Streck, Omaha, NE), CellSave, Heparin and EDTA (ethylenediaminetetraacetic acid) Vacutainer tubes were used for extraction of circulating cell free DNA from HNV. Plasma was separated from whole blood by two sequential centrifugations (each 2000*g*, 10 min) followed by upper phase plasma removal and stored at −80°C in 2 ml aliquots (Rothwell *et al*., 2016). Cell-free DNA (cfDNA) was isolated from upto 4 ml of double spun plasma using the QIAsymphony in conjunction with Circulating DNA Kit (Qiagen, Hilden, Germany). Following isolation, cfDNA yield was quantified using the TaqMan^®^ RNase P Detection Kit (Life Technologies) as per manufacturer's instructions (Rothwell *et al*., 2016). Germline DNA (gDNA) was isolated from EDTA whole blood using QIAmp Blood Mini Kit (Qiagen, Hilden, Germany) as per manufacturer's instructions.

### 2.4 Mutation calling using MuTect software

MuTect (Cibulskis *et al.*, 2013), was run with default parameter settings as per the GATK best practices recommendations (https://software.broadinstitute.org/gatk/best-practices/mutect1.php) with two additional parameters: ‘-dt NONE -min_qscore 20’.

## 3 Results

We first evaluated *PEC* using the reference DNA dataset used by Newman *et al.* to evaluate *iDES* (Newman *et al.*, 2016). These data were generated by mixing acoustically shorn HD500 genomic DNA (Horizon Diagnostics) with cfDNA from a healthy donor. The resultant data incorporate a set of known clinically relevant variants with a wide range of allele frequencies (AF) (0.94% ≤ AF ≤ 32.5%). 4 technical replicates with 5% simulated ctDNA content were generated.

Comparisons were made between *PEC*, the *Background-Polishing* step of *iDES* and *MarkDuplicates*, using each algorithm as a pre-processing step prior to mutation calling. Since no matching genomic DNA was available, we were unable to use the standard GATK pipeline for mutation calling, and instead adopted the approach used by Newman and colleagues: a Poisson model (Newman *et al.*, 2016), was used to define the theoretical minimum number of variant alleles required to detect ctDNAs with 95% confidence. Loci where the VAF exceeded this detection limit were called as variants. Target sequences covered a total of 302 620 nucleotides. These encompassed 239 known SNVs previously shown to be clinically relevant in NSCLC (Newman *et al.*, 2016) that were not present in the data, alongside 26 True Positive (TP) SNVs that were present.

**Fig. 2.**
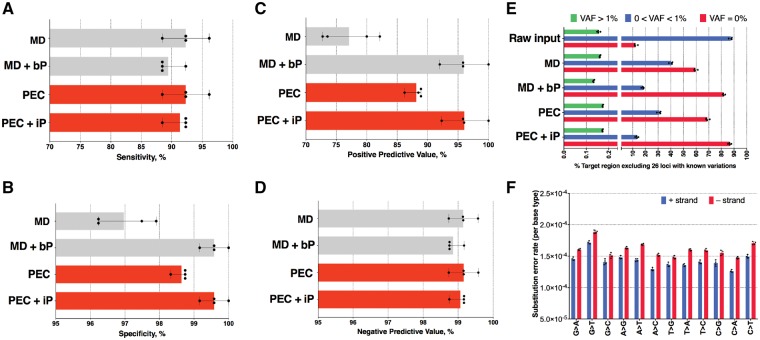
Accuracy of each pipeline using Dataset 1. Each point represents an individual dataset. (**A**) Sensitivity %, (**B**) Specificity %, (**C**) Positive predictive value %, (**D**) Negative predictive value %, (**E**) % Target loci (excluding 26 loci with known variations) with VAF = 0, 0 < VAF < 1% and VAF > 1% before and after error suppression. (**F**) Substitution error rate estimated from *PEC-DeDuplicates* and summarized by nucleotide, and MD represents the error suppression by *MarkDuplicates*. MD + bP represents the error suppression by *MarkDuplicates* and *Background-Polishing*. PEC represents the error suppression by *DeDuplicates*. PEC + iP represents the error suppression by *DeDuplicates* and *Intrinsic-Polishing*

Data were evaluated using the widely adopted GATK pipeline, configured according to the GATK ‘best practices recommendations’ (see Section 2). The performance of the standard pipeline was compared to that achieved by substituting the *MarkDuplicates* step with *PEC. PEC* exhibited improved specificity and positive predictive value over *MarkDuplicates*, while matching the specificity and positive predictive value of the *iDES Background-Polishing* algorithm (Fig. 2A–D). Importantly, *PEC* achieves these improvements without the need to reference an external HNV dataset. As expected, PEC also suppressed more background errors in regions known to be absent of SNVs compared to *Extrinsic-polishing* (Fig. 2E and Supplementary Figs S2–S3). These errors included a disproportionate number of G > T and C > T transitions (Fig. 2F), as previously reported (Newman *et al.*, 2016).

We further evaluated PEC using a second reference dataset generated by mixing acoustically shorn reference DNA derived from H466 cells with cfDNA from a healthy normal donor (see Section 2). Three samples were generated at different concentrations along with 100% HNV cfDNA and 100% H466 DNA were also sequenced and analyzed using the unmodified GATK best practices workflow (McKenna *et al.*, 2010), in order to identify SNVs specific to the cell line. These were then used as a ‘gold standard’ True Positive set for subsequent analyses. In total, 109 variant alleles were selected as TP SNVs with VAFs > 25% (Supplementary Fig. S4). Having established a ground truth for evaluation, comparisons were then made between *PEC* and *MarkDuplicates*, using each algorithm as a pre-processing step prior to mutation calling using MuTect (Cibulskis *et al.*, 2013).

**Fig. 3.**
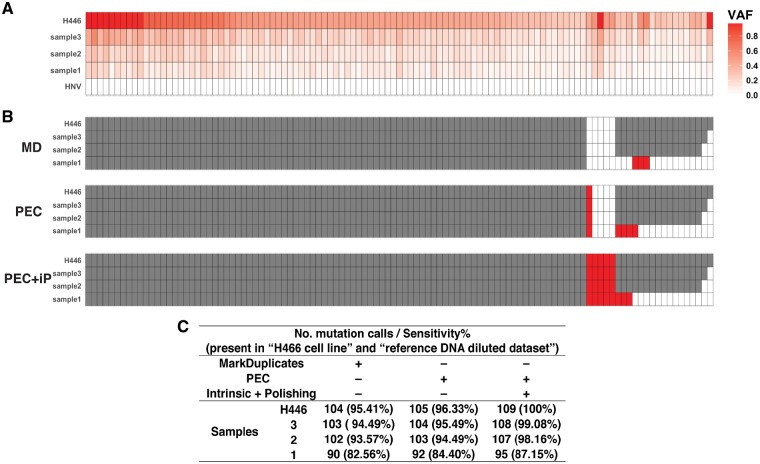
Comparison of the sensitivity of each pipeline using Dataset 2. 5 dilutions were generated (simulating 0, 5, 10, 20, 100% ctDNA). Sensitivity was calculated using 109 True Positive loci with VAFs > 25% in the 100% H446 sample. In all panels, each column corresponds to a target locus. (**A**) VAFs of True Positive loci for each sample data (see also Supplementary Fig. S4). (**B**) Mutation calls from each pipeline using MuTect software. White: no mutation call, grey: called by all pipelines, red: called correctly by some pipelines. MD: *MarkDuplicates* pipeline. PEC: PEC *DeDuplicates* pipeline. PEC + iP: PEC *DeDuplicates* and *Intrinsic-Polishing* pipeline. (**C**) Number of mutation calls and sensitivity. In (A) and (B), each column corresponds to a known SNV and each row represents a simulated ctDNA sample

As before, PEC exhibited increased sensitivity over *MarkDuplicates* at all dilutions (Fig. 3). Importantly, 4 SNVs were only called when *Intrinsic-Polishing* was employed. In all four cases, these loci had low, but significant VAF (>2%) in the 100% HNV cfDNA sample (Supplementary Fig. S5); these variant alleles in the normal input were removed by *Intrinsic-Polishing*, leading to the correct mutation calls at these loci. Finally, we compared the error correction patterns between the two datasets, to reveal substantial differences between the two datasets (Supplementary Fig. S6). Background polishing algorithms that take these error patterns into account must therefore be able to model different error patterns for each dataset presented. The intrinsic polishing approach described here is therefore particularly appealing since it does not require access to multiple external reference datasets in order to learn these patterns.

Taken together, these data demonstrate that when *PEC* is incorporated into the GATK best practices variant calling pipeline it leads to improved sensitivity and specificity when used as a direct replacement of *MarkDuplicates.* It achieves similar performance to the *Background-Polishing* approach used by *iDES*, even though it does this without recourse to a reference HNV dataset. Since PEC’s contribution is to correct errors by improving the de-duplication step, it is likely to have general utility beyond the GATK best practices pipeline. However, careful validation will be required before adopting it in each alternate setting. Thus, although the data we present here demonstrates the utility of *PEC* in the context of ctDNA data, we expect it to be widely applicable to other NGS datasets including those arising from single cell analyses.

## Supplementary Material

bty1004_Supplementary_Data
