# Supplementary material for: In silico error correction improves cfDNA mutation calling
Source: Bioinformatics. 2018 Dec 6;35(14):2380–5. doi: 10.1093/bioinformatics/bty1004 (PMC6612818; doi:10.1093/bioinformatics/bty1004)
Supplement: bty1004_Supplementary_Data [file bty1004_supplementary_data.docx]

**Supplementary Figures**

**
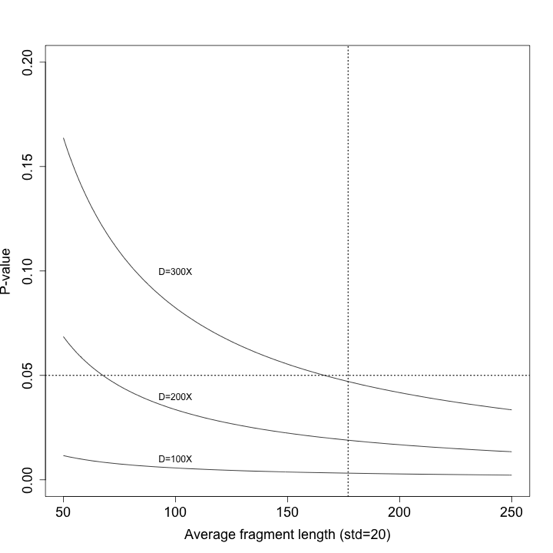
**

**Fig. S1:** Estimated probability of cfDNA fragment collisions at different fragment lengths and cfDNA fragment depths.


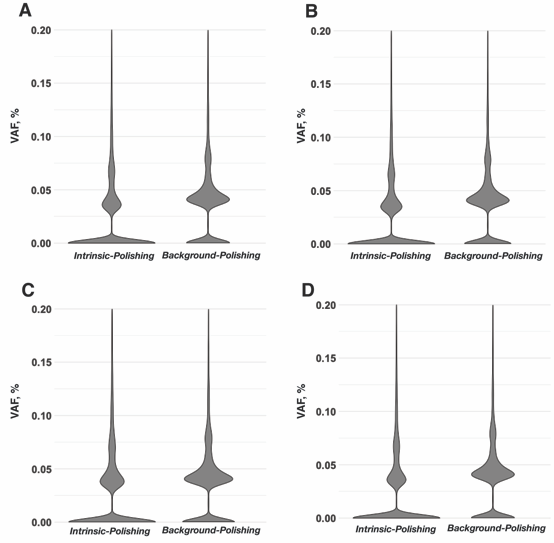


**Fig. S2**: Distribution of VAFs % by Intrinsic-Polishing and Background-Polishing for 4 samples of HD500 reference DNAs^12^: (a) SRR3503022 (b) SRR3503023 (c) SRR3503024 (d) SRR3503025. 26 loci with known mutational variations were excluded and loci with AF < 1% were collected.


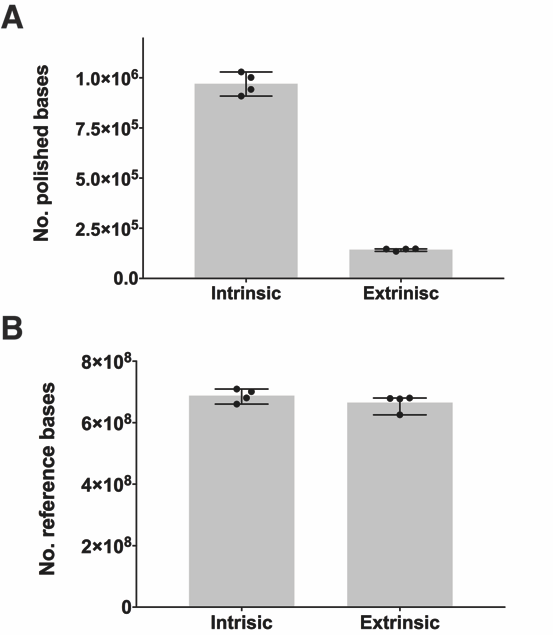


**Fig. S3**: Number of polished error bases and reference bases by *Intrinsic* and *Extrinsic* polishing. (a) Number of polished error bases. (b) Number of bases as reference alleles after polishing step.


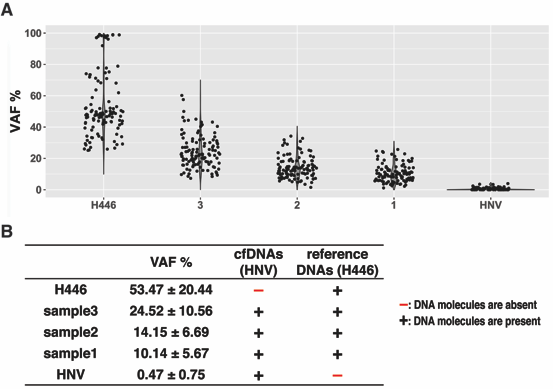


**Fig. S4**: The effective amount of the reference DNAs from H466 cell line in 5 samples of H466 reference DNAs. (a) 5 samples of data were created by mixing cfDNAs from HNV and reference DNAs. Each dot represents the VAFs of each genomic locus (b) Effective amount of reference DNAs from H446 cells. The HNV sample only contains the cfDNAs from HNV, which was used as normal input to MuTect software for mutation calling. H446 sample contains only reference DNA from H446 cells, in which 109 loci with VAF > 25% were selected as TP variants for sensitivity calculations.


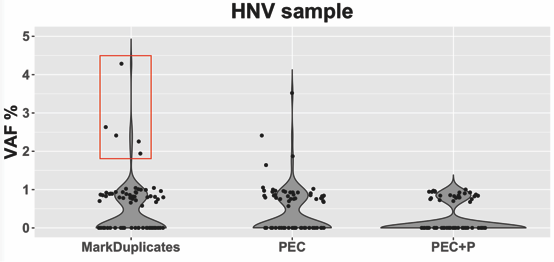


**Fig. S5**: Distribution of VAF % in the HNV sample. Each dot corresponds to each genomic location of 109 selected TP variants. MD: error suppression by *MarkDuplicates*. PEC: error suppression by *DeDuplicates*. PEC + P: error suppression by *DeDuplicates* and *Intrinsic-Polishing*. The 5 points within the red box correspond to 5 FN mutation calls by the MD pipeline. These were successfully removed by *Intrinsic-Polishing*.


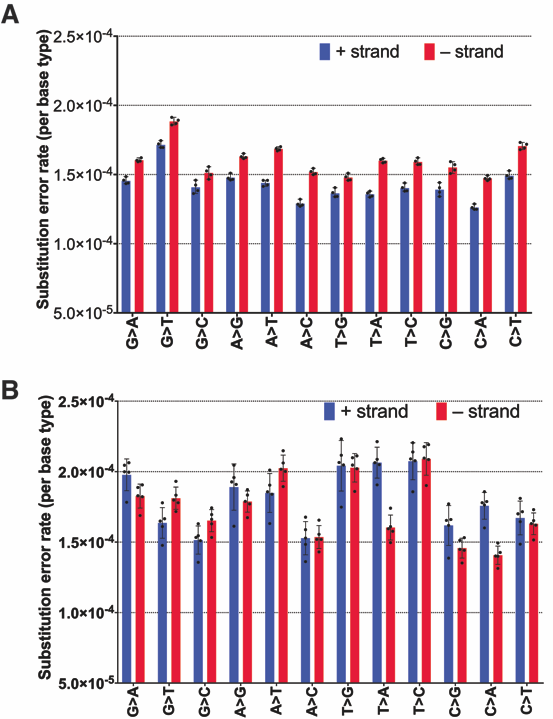


**Fig. S6**: Substitution error rate estimated from *PEC-DeDuplicates* and summarised by nucleotide. (a) Dataset 1 (b) Dataset 2.
